# A commentary on “Individualized blood pressure regulation and acute kidney injury in older patients having major abdominal surgery: a pilot randomized trial”

**DOI:** 10.1097/JS9.0000000000003230

**Published:** 2025-08-23

**Authors:** Fu-Shan Xue, Dan-Feng Wang, Xiao-Chun Zheng

**Affiliations:** a Department of Anesthesiology, Shengli Clinical Medical College of Fujian Medical University, Fuzhou University Affiliated Provincial Hospital, Fujian Provincial Hospital, Fuzhou, China; b Department of Anesthesiology, Beijing Anzhen Hospital, Capital Medical University, Beijing, China

*Dear Editor*,

By conducting a single-center pilot randomized controlled trial in a total of 179 elderly patients who underwent elective laparoscopic cancer surgery with general anesthesia, Pang *et al*^[1]^ demonstrated that individualized blood pressure management during surgery significantly decreased the occurrence of intraoperative hypotension, with a statistically no-significant but clinical meaningful reduction in postoperative acute kidney injury (AKI). Before accepting their conclusions, we believe that several issues about the methodology and results of this study warrant further clarification and discussion.

First, all participants were elderly patients undergoing major laparoscopic cancer surgery, with a mean age of ≥68 years. Pang *et al*^[1]^ did not assess preoperative nutritional status, hemoglobin and serum protein levels. Available literatures indicate that preoperative malnutrition, anemia and hypoalbuminemia are frequent among elderly patients undergoing cancer surgery and have been identified as independent risk factors for development of AKI after non-cardiac surgery^[2-4].^ Most important, median surgical duration was 220 min or longer, but Pang *et al*^[1]^ did not specify whether same intra-abdominal pressure was applied in all patients. It is reported that an intra-abdominal pressure >10 mmHg may decrease renal blood flow and cause renal dysfunction and transient oliguria^[5]^. In addition, it was also unclear whether the two groups were comparable with respect to duration of intraoperative pneumoperitoneum and exposure index (product of intra-abdominal pressure and inflation time), albeit they have been associated with an increased risk of AKI after abdominal surgery^[6,7]^. We are concerned that any between-group imbalance in above-mentioned confounders would have significantly biased primary outcome.

Second, Pang *et al*^[1]^ described that intraoperative blood pressure target in the routine care group was designed to keep a SBP ≥90 mmHg and a MAP ≥65 mmHg, according to 2019 perioperative quality initiative consensus statement on intraoperative blood pressure, risk and outcomes for elective surgery^[8]^. In fact, this consensus statement clearly indicates “*even brief durations of SBP <100 mmHg and MAP <60–70 mmHg are harmful during non-cardiac surgery*,” as they are associated with myocardial injury, AKI and death^[8]^. To ensure perioperative safety of patients with a high risk of vital organ injury^[2]^, it is frequently recommended that intraoperative blood pressure should be maintained at upper allowable range^[9]^. Thus, we believe that in Pang *et al*’s study, intraoperative blood pressure target in the routine care group should be to keep a SBP ≥100 mmHg and a MAP ≥70 mmHg, rather than a SBP ≥90 mmHg and a MAP ≥65 mmHg. Most important, hypertensive patients accounted for 34–39% of study population. The design of intraoperative blood pressure target considered baseline blood pressures in the individual treatment group, but not in the routine care group. As pneumoperitoneum may promote development of AKI by reducing renal blood flow and glomerular filtration rate^[5]^, an important question worth considering is if anesthesiologists really allow intraoperative blood pressure to be maintained at lower allowable range for elderly hypertensive patients with major laparoscopic surgery. The robust evidence indicated keeping intraoperative blood pressure close proximity to baselines may improve postoperative outcomes^[9]^. Especially, a 5-mmHg difference of intraoperative MAP target can change occurrence of AKI after major abdominal surgery in elderly hypertensive patients^[10]^. Due to above design issues, the routine care group compared with the individual treatment group had a lower mean intraoperative MAP, a higher incidence of intraoperative hypotension and longer cumulative durations of intraoperative MAP <65 and <60 mmHg, resulting an increased risk of postoperative AKI^[8]^. Thus, we argue that findings of this study may not be extrapolated into elderly population who undergo major laparoscopic surgery with clinical managements that differ from research protocol of Pang *et al*.

Third, overall incidence of postoperative AKI demonstrated a reduction of 28% with individualized blood pressure management, but this group had one patient with stage 3 AKI and one with renal replacement therapy, indicating that critical AKI, which are more associated with increased short- and long-term adverse outcome^[11]^, occurs. Nevertheless, critical AKI did not occur in the routine care group. Patients receiving individualized blood pressure management had a shorter postoperative mechanical ventilation duration and a faster recovery of bowel function, but Pang *et al* did not state if the two groups were comparable in terms of postoperative sedatives opioid analgesics. Furthermore, patients receiving routine care had a trend of shortening median length of hospital stay by 1 day. Thus, we do not agree with Pang *et al*’s conclusion that reduction of postoperative AKI with individualized blood pressure management is clinically meaningful.

Finally, we ensure that our article is compliant with the TITAN Guidelines 2025, as shown in TITAN Guideline Checklist 2025^[12]^.

## Data Availability

All data are available.
